# An Ultrasensitive miRNA-Based Genosensor for Detection of MicroRNA 21 in Gastric Cancer Cells Based on Functional Signal Amplifier and Synthesized Perovskite-Graphene Oxide and AuNPs

**DOI:** 10.3390/bios13020172

**Published:** 2023-01-22

**Authors:** Payam Shahbazi-Derakhshi, Elham Mahmoudi, Mir Mostafa Majidi, Hessamaddin Sohrabi, Mohammad Amini, Mir Reza Majidi, Aligholi Niaei, Nima Shaykh-Baygloo, Ahad Mokhtarzadeh

**Affiliations:** 1 Department of Analytical Chemistry, Faculty of Chemistry, University of Tabriz, Tabriz 5166-616471, Iran; 2 Department of Biology, Faculty of Science, Urmia University, Urmia 5756-151818, Iran; 3 Immunology Research Center, Medical Science University of Tabriz, Tabriz 5166-15731, Iran; 4 Catalyst and Reactor Research Lab, Department of Chemical & Petroleum Engineering, University of Tabriz, Tabriz 5166-616471, Iran; 5 Department of Chemical Engineering, Amirkabir University of Technology, Tehran 1591-634311, Iran

**Keywords:** electrochemical genosensing, miRNA-21, gastric cancer, perovskite, gold nanoparticles

## Abstract

In the present research work, the state-of-art label-free electrochemical genosensing platform was developed based on the hybridization process in the presence of [Fe(CN)_6_]^3−/4−^ as an efficient redox probe for sensitive recognition of the miRNA-21 in human gastric cell lines samples. To attain this aim, perovskite nanosheets were initially synthesized. Afterward, the obtained compound was combined with the graphene oxide resulting in an effective electrochemical modifier, which was dropped on the surface of the Au electrode. Then, AuNPs (Gold Nano Particles) have been electrochemically-immobilized on perovskite-graphene oxide/Au-modified electrode surface through the chronoamperometry (CA) technique. Finally, a self-assembling monolayer reaction of ss-capture RNA ensued by the thiol group at the end of the probe with AuNPs on the modified electrode surface. miRNA-21 has been cast on the Au electrode surface to apply the hybridization process. To find out the effectiveness of the synthesized modifier agent, the electrochemical behavior of the modified electrode has been analyzed through DPV (differential pulse voltammetry) and CV (cyclic voltammetry) techniques. The prepared biomarker-detection bioassay offers high sensitivity and specificity, good performance, and appropriate precision and accuracy for the highly-sensitive determination of miRNA-21. Different characterization methods have been used, such as XRD, Raman, EDS, and FE-SEM, for morphological characterization and investigation of particle size. Based on optimal conditions, the limit of detection and quantification have been acquired at 2.94 fM and 8.75 fM, respectively. Furthermore, it was possible to achieve a wide linear range which is between 10^−14^ and 10^−7^ for miRNA-21. Moreover, the selectivity of the proposed biosensing assay was investigated through its potential in the detection of one, two, and three-base mismatched sequences. Moreover, it was possible to investigate the repeatability and reproducibility of the related bio-assay. To evaluate the hybridization process, it is important that the planned biomarker detection bio-assay could be directly re-used and re-generated.

## 1. Introduction

MicroRNAs (miRNAs) are non-coding ribonucleic acids that are evolutionarily conserved and have a length of 18–22 nucleotides [1,2,3]. miRNAs control gene expression after transcription by inhibiting target mRNAs or inhibiting their translation [4,5,6]. miRNA regulates a variety of cellular processes such as cell proliferation, cell differentiation, immunogenesis, insulin secretion, and cell apoptosis [7,8,9,10,11]. As yet, it was possible to identify an enormous variety of miRNAs in microorganisms, plants, animals, and humans (over 4000 miRNAs) (http://www.mirbase.org/. accessed on 1 October 2022), and it was calculated to be more than target 30% of the human genome [12,13]. miRNAs are abundant mainly in tissues and body fluids, including cerebrospinal fluid, breast milk, tears, pleural fluid, peritoneal fluid, saliva, urine, and plasma [14]. By interfering with intracellular messenger RNA (mRNA), either directly by mRNA cleavage or indirectly by translational suppression, miRNAs regulate genes. miRNAs are now known as clinical therapies, where aberrant expression of miRNAs is directly related to cancer, and miRNAs play a significant role in cancer development, progression, and metastasis [15,16]. Thus, cancer antagomir and tumor suppressor miRNAs can be applied as efficient treatments for cancer [17,18]. Due to numerous diseases and the incidence of various cancers, and the dangers for humans and other organisms, rapid and highly sensitive identification of miRNA-21 biomarkers is of great importance [13,19,20].

Clinical determination of miRNAs is challenging because of the enormous apparatuses, high expenses, difficulty of sample preparation and tedious tool performance, the necessity for skilled operators, and the time-consuming process. Moreover, in most cases, using clinical imaging techniques, only large tumors can be seen, and therefore, most cancers are diagnosed at an intermediate or advanced stage, and the probability of being diagnosed with cancer at an acceptable stage of treatment is less [21,22,23]. For example, the limited linear quantification range, imperfect sensitivity for long sequence homogeneity, and the inability to detect new miRNAs and determine the abundance of miRNAs are disadvantages of the miRNA microarray technique. Although qRT-PCR has a very high sensitivity and dynamic range, its main limitation is its high cost and the need for a computing infrastructure to analyze and interpret data. Nevertheless, miRNA determination needs a greatly sensitive method development for assessing very low levels of sample in the bloodstream and additionally requires to be very selective and comply with multiplexing capabilities of a diagnostic test, cost-effectiveness, and minimum sampling volume requirement [24,25,26].

Sensing assays have attracted much attention due to fast, selective, sensitive, and low-cost analytical devices, including two elements of biological recognition element for capturing target and converter miRNAs detection reaction signal into a measurable signal [27,28]. Based on the type of transducers, biosensors can be classified into several major groups, including optical, thermal, piezoelectric, and electrochemical sensors [29,30]. Detection in optical biosensors is based on the measurement of light absorbed or emitted as a result of a biochemical reaction between an analyte and a biological receptor. Optical biosensors are based on various optical methods such as absorption, fluorescence, luminescence and surface plasmon resonance [31,32]. Thermal sensors, as the name suggests, it is the measurement of thermal changes that have occurred on the surface of the biochemical detector. Many biochemical reactions involve a change in enthalpy that can be measured using sensitive thermostats [29]. Piezoelectric sensors can measure mass change and, thus, biomolecular interaction [33]. Each of these methods has its advantages and disadvantages [34,35]. Compared to other methods, electrochemical methods are low-cost, higher-sensitivity, selective, and portable decomposition techniques that are widely used in the fields of environment, agriculture, medicine, and food; a method that can take the target analyzes and convert the biological signals into electrical signals, respectively [36,37,38,39,40]. Due to significant advances in nanotechnology and related sciences, the increase in electrochemical sensing platforms by modifying the electrode surface based on nanomaterials has made it possible to improve sensitive sensors as well as electrochemical selectivity [41,42,43]. Graphene is a material with unique properties that have high intrinsic mobility and high thermal conductivity, good optical and electrical conductivity for applications such as transparent conductive electrodes, among many other noteworthy potential applications [44]. On the other hand, synthesized perovskite compounds are known as attractive nanomaterials for use in analytical studies [45,46,47]. These oxides are known as magic catalysts in different applications due to their excellent physical and chemical properties, flexible structure, and catalytic potential. The catalytic activity and application of perovskites can be modified by doping with various elements of the periodic table. Perovskite/graphene oxide nanocomposite has been used as an innovative modifier to enhance the electrochemical signal whose properties and benefits can be attributed to its conductivity, unique physical, chemical, optical and catalytic properties, as well as fast and easy synthesis [48,49,50]. Being with the environment, narrow size distribution and feasibility at low temperatures [37,51,52].

In this study, an electrochemical oligonucleotide genosensor was developed to detect and measure miRNA-21. To achieve this goal, multilayer nanocomposites based on perovskite-graphene oxide were initially synthesized. The resulting nanocomposite was immobilized on the surface of the working electrode by the dropping method. Next, a single-stranded RNA probe (ss-capture RNA) was immobilized on the electrode. The interaction of the target RNA probe (miRNA-21) with the ss-capture RNA affects the electrochemical behavior of the electrode or interphase function because of miRNA hybridization to signal, electrochemically active reporter changes in electrode properties. As a result, with various analytical features, it is possible to identify and detect miRNA-21 in the human gastric cell line sample.

## 2. Experimental

### 2.1. Apparatus and Instrumentation

To perform the biosensing and electrochemical analysis, the Galvanostat/Potentiostat Autolab PGSTAT 30 (Eco Chemie B.V., Utrecht, The Netherlands) has been applied. A cell containing three electrodes has been used to perform the electrochemical analysis. The modified and bare gold electrodes have been utilized as working electrodes (d = 2 mm). The reference electrode was a saturated calomel electrode (SCE and Hg_2_Cl_2_), and the auxiliary electrode was a Pt wire electrode in a KCl solution.

Azar electrode Co. (Urmia, Iran) has been selected to buy all electrodes. For characterization purposes, the Bruker instruments, Germany, model Aquinox 55 has been selected to obtain the Fourier-transform infrared (FT-IR) spectra. To explain the surface morphology, the energy-dispersive X-ray analysis (EDS) and scanning electron microscopy (SEM) have been examined on a MIRA3 FEG-SEM TESCAN developed in the Czech Republic. Siemens diffractometer (D500 S) has been used to examine the composition of synthesized X-ray diffraction (XRD) and perovskite/graphene oxide analysis, and it has been applied at room temperature, where the scan range of 2θ was from 5 to 70°, and Cu- Kα radiation was at 35 kV. StepOnePlus real-time PCR (Applied Biosystems, Foster City, CA, USA) was used to carry out gene expression analysis.

### 2.2. Reagents and Materials

Starting materials for La_0.8_Sr_0.2_Cu_0.7_Mn_0.3.3_/SiO_2_ (LSCMO_3_/SiO_2_) were La(NO_3.3_·6H_2_O, Cu(NO_3.2_·3H_2_O, and glycine (99%), purchased from Samchun Pure Chemical Company, Mn(NO_3.2_·4H_2_O, Sr(NO_3.2_, were purchased from Merck and de-ionized water. Potassium chloride (KCl), hydrogen tetrachloroaurate (III) hydrate (HAuCL_4_·3H_2_O), potassium ferrocyanide K_4_Fe(CN)_6,_ and potassium ferricyanide K_3_Fe(CN)_6_ were purchased from Sigma-Aldrich (Winston Park Drive Oakville, ON, Canada). Merck (Rahway, NJ, USA) Chemicals Co. has been selected to obtain Hydrogen peroxide solution (H_2.2_) 30%, acetone (CH_3_COCH_3_), hydrochloric acid (HCl), sulfuric acid (H_2_SO_4_) (0.5 M), sodium acetate (CH_3_COONa), mercaptoethanol (HS(CH_2.6_OH), DLdithiotrietol (DTT), ethanol and all other demanded supplies. A ferrocyanide/ferricyanide solution including 0.01 M of K_4_Fe(CN)_6_/K_3_Fe(CN)_6_ (1:1) and 0.1 M KCl has been prepared to obtain the effective electrochemical examination of the working electrode surface areas. Piranha solution with hydrogen peroxide, water, and concentrated sulfuric acid in a 1:1:1 (*v*/*v*) ratio has been obtained and instantly utilized to maintain its reactivity. By mixing 500 mM of DTT and 10 mM of sodium acetate at pH = 5.2 and kept at 4 °C, a DTT solution has eventually been obtained. Double-distilled DEPC water has been used in all experimental steps. Solvents utilized for experimental purposes were used without any purification and had an analytical grade.

Bioneer Co. (Daejeon, Republic of Korea) has been selected to purchase applied RNA oligonucleotides with sequences in Table 1. Moreover, stock solutions of the oligonucleotide have been diluted with 0.1 M tris-HCL buffer at pH = 7.4. Pasteur Institute (Tehran, Iran) has been selected to obtain human gastric cancer cell lines. The cells were transfected with anti-miR-21 (GenePharma Co., Shanghai, China). According to supplied procedures, total RNA has been extracted using the GeneAll Trizol RNA extraction kit (Daejeon, Republic of Korea). To quantify miRNAs expression, 1 μg of extracted RNA was initially converted to complementary RNA (ss-RNA) synthesis by the Universal cDNA Synthesis miRCURYLNATM kit (Exiqon, Copenhagen, Denmark).

### 2.3. Nanocomposite Preparation Steps

#### 2.3.1. Preparation of Perovskite and Chemically-Synthesis of Graphene Oxide (GO)

To synthesize the La_0.8_Sr_0.2_Cu_0.7_Mn_0.3.3_/SiO_2_ perovskite, denoted as LSCMO-S, the sol-gel combustion method. Firstly, SiO_2_ was dispersed in deionized water with continuous stirring. In the next step, the stochiometric amounts of metal nitrates were added to the suspension and heated up to 85 °C. At this temperature, the glycine was added, and the NH_3_·H_2_O was to adjust the pH of the solution to 6. After about 2 h, a gel was formed. In this step, the temperature was increased suddenly to 250 °C until combustion happened and a black powder was obtained. Finally, the obtained black powder calcinated at 700 °C for 6 h [53,54].

For the synthesis of graphene oxide, denoted as GO, Hummer’s method [55]. Sulfuric acid (H_2_SO_4_ 98%) was stirred in the ice bath, sodium nitrate and graphite (2.5/5) were added slowly in 10 min with vigorous stirring, and the solution was stirred for 1 h in the ice bath until a viscose solution was obtained. Potassium permanganate was very carefully poured into the solution for 2 h, and then the ice bath was removed. The solution was left at room temperature overnight, and a light brown suspension was obtained. Deionized water was added in 1 h, and the temperature increased to 98 °C and refluxed for 24 h. the solution cooled at room temperature, then an H_2.2_ solution was added and stirred at room temperature, and the end of the process, HCl solution was added and stirred for 24 h. the acid-washed solution was washed and centrifuged till it obtained pH = 7. The solution was dried overnight at 90 °C.

#### 2.3.2. Preparing Perovskite/Graphene Oxide Nanocomposite

The catalyst powders (perovskite/graphene oxide nanocomposite) were crushed by a physical method for 1 h (denoted as LSCMO-S/GO). The complete dispersion of synthesized perovskite among graphene oxide sheets is confirmed by the obtained result. Then the powders were dispersed in NMP (N-Methyl-2-pyrrolidone) (11 g·L^−1^) and sonicated in an ultrasonic bath for about 2 h. The prepared solution was effectively used for the modification of the electrode surface.

### 2.4. Fabricating Electrochemical Genosensing Assessment

For obtaining the newest surface of the Au electrode before coating and performing electrochemical analysis, alumina-saturated woven glasses were used to polish it, and then, it took 20 min to float it in Piranha solution. Afterward, a water/acetone mixture was used to float the electrode for 20 min, where the volume ratio was 1:1 (*v*/*v*), and it was then washed with double distilled water. During the first step and to modify the Au electrode surface, the synthesized perovskite/graphene oxide compound has been utilized as an efficient substrate. By so doing, it was possible to dissolve a specified quantity of the synthesized compound in an NMP solution and immobilize it on the surface of Au electrodes using the dropping method. To improve the electrochemical signal in the best possible manner, AuNPs (synthesized gold nanoparticles) have been immobilized on the modified Au electrode surface using the CA method accompanied by complete drying and stabilization of the surface modifiers.

**Figure 1:**
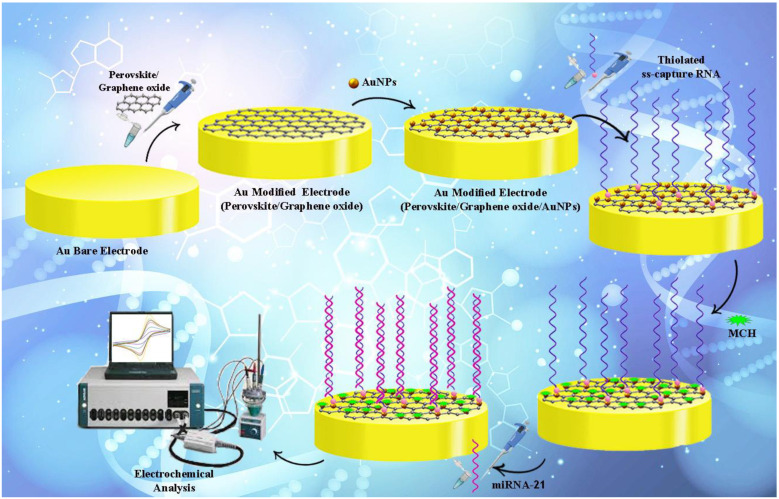
The fabrication process of the electrochemical genosensing platform.

Ss-capture RNA has been treated with DTT (dithiothreitol) to fabricate the RNA-based genosensor. Dithiothreitol (DTT) has been considered an efficient protecting mediator for thiolated ss-capture RNA, which prevents thiol group oxidation. It is considered a substantial mediator and can be used in clinical medicine, biochemistry, peptide-protein reaction, and chemistry [56]. When oxygen exists in the solution, the end sulfur atoms related to a thiolated ss-capture RNA have dimerization affinity. The effectiveness of significant immobilizing reactions, such as the immobilization of RNA on the Au electrode in the biosensing assay, is diminished by forming dimer compounds. According to such research, a 10-μL DTT has been added to a 10-μL ss-capture RNA solution. Based on the next step, the acquired solution was mixed by a vortex and located for 30 min at 25 °C. Afterward, the surface of AuNPs/perovskite/graphene oxide/Au electrode was used for dropping the solution, and it took 2 h for incubation to be performed at 4 °C. Finally, self-assembling monolayer reaction of ss-capture RNA ensued on the Au-modified electrode surface. At this stage, the electrode was rinsed with double distilled water, and it took 20 min to incubate the ss-capture RNA-modified genosensor by MCH (mercaptohexanol) at 25 °C. As a short-chain thiol with a hydrophilic hydroxyl group, mercaptoethanol (MCH) is frequently co-assembled with probe RNA to make probe RNA erect on an Au-modified electrode and reduce nonspecific adsorption of probe RNA. RNA hybridization performance can be improved by co-assembly of MCH with probe RNA on Au [57,58]. In another word, nonspecific interactions are displaced by MCH between the gold and RNA, and SAM (self-assembled monolayer) is formed, which withstands nonspecific adsorption of target RNA. The remaining gold sites have been filled by MCH immobilization, and a well-aligned ss- RNA monolayer has been formed. Blocking non-reacted AuNPs with an RNA probe was the purpose of this step. At the next stage, the take-up of the miRNA-21 is significantly increased by MCH. At the final stage, miRNA-21 was cast on the Au electrode surface to apply the hybridization process. As a result, the solution containing KCl and [Fe(CN)_6_]^3−/4−^ has been selected to perform the electrochemical analysis. The fabrication process of the electrochemical genosensing platform is represented in Figure 1.

### 2.5. Electrochemically Detecting miRNA-21

After successfully fabricating the ss-capture RNA/AuNPs/perovskite/graphene oxide/Au electrode bioelectrode, incubation for 50 min was used to test various concentrations of miRNA-21, which has a range of 10 fM–100 nM. It was possible to hybridize ss-capture RNA to the target miRNA-21 with strong affinity and produce concentration-dependent signals that have electrochemically been calculated by DPV and CV assay.

### 2.6. Real Samples

#### 2.6.1. Cell Culture

The Pasteur Institute (Tehran, Iran) has been selected to obtain the human gastric cancer cell lines, including MKN-45, KATO III, and AGS cell lines, and RPMI 1640 medium has been used to culture them, and then they have been enriched with 10% FBS and contained 1% antibiotics (100 Unit/mL penicillin, and 100 μg/mL streptomycin) at an incubator providing 37 °C, 5% CO_2_ and 95% humidity. Subsequently, qRT-PCR was performed to evaluate miRNA expression levels in mentioned cell lines for the selection of the target cell line with higher expression of miRNA 21.

#### 2.6.2. MicroRNA Transfection

According to the protocols supplied (TC = 12.5 ms and Volts = 160 v), the AGS cell line was transfected with anti-miRNA-21 and FITC-conjugated controls (GenePharma Co., Shanghai, China) in the amount of 40 pmol with the help of Gene Pulser electroporation system (Bio-Rad, Hercules, CA, USA). 2 × 10^5^ transfected cells have been seeded into six-well plates and cultured for 24, 48, and 72 h. Subsequently, qRT-PCR was performed to evaluate miRNA expression levels in the transfected group and estimate the optimum time and dose for anti-miRNA 21 transfection.

#### 2.6.3. Quantitative Real Time-Polymerase Chain Reaction (qRT-PCR)

According to supplied procedures, the GeneAll Trizol RNA extraction kit (Daejeon, Republic of Korea) has been used to extract total RNA. To quantify miRNA expression, the Universal cDNA Synthesis miRCURYLNATM kit (Exiqon, Copenhagen, Denmark) has been used to initially convert 1 μg of extracted RNA to the synthesis of cDNA (complementary DNA). BioFACT™ 2X Real-Time PCR Master Mix (Daejeon, Republic of Korea) in the StepOnePlus Real-Time PCR System (Applied Biosystems, Foster City, CA, USA) has been used to measure miRNA-21 at target cells and evaluation of miRNA 21 expression at anti-miRNA 21 treated cells. For normalizing miRNA 21 expression, U6 was used as the internal control. The primer sequences were designed and blasted using the NCBI primer blast online tool (www.primerdesigntools.com, accessed on 28 May 2021). Each reaction was repeated three times, and the 2-ΔΔCT method was used to measure the relative expression levels of genes.

## 3. Results and Discussion

### 3.1. Surface Morphology of the Gold Electrode

#### 3.1.1. Field Emission Scanning Electron Microscope

**Figure 2:**
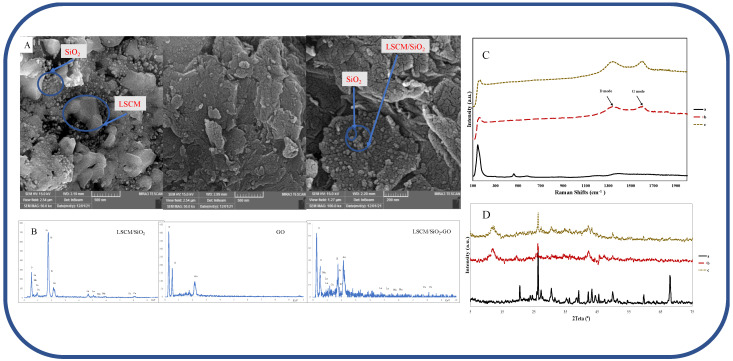
(**A**) SEM and (**B**) EDS results of LSCMO-S, GO, and LSCM-S/GO, (**C**) Raman spectra of LSCMO-S (a), GO (b), LSCMO-S/G (c), (**D**) XRD results of LSCMO-S (a), GO (b), and LSCMO-S/GO (c).

The morphology of LSCMO-S, GO, and LSCMO-S/GO was investigated by scanning electron microscopy. The SEM image of the synthesized perovskite is represented in Figure 2A. In Figure 2, two different phases of LSCM (indistinctive shape) and SiO_2_ spherical shapes with an average diameter of about 30 nm were observed, and silica particles were embedded into the LSCM matrix that was validated with the Scherrer equation. In some segments (as seen in the SEM), the amorphous SiO_2_ blocked the diffusion of metal ions during the combustion reaction, which is undesirable for perovskite formation. The morphology of GO shows a typically synthesized graphene oxide and the composite of LSCMO-S/GO (containing 10% LSCMO-S), suggesting that the presence of the perovskite does not modify graphene oxide’s pattern and the morphology retains well.

#### 3.1.2. Energy-Dispersive X-ray Spectroscopy (EDS)

EDS (Figure 2B) investigation is carried out to determine the elemental composition of the material synthesized at a voltage of 15 kV, and a Si detector has been used to capture the spectrum, which is a characteristic X-ray. The LSCMO-S comprises 38% silicon and 37% oxygen, which indicates that the main elements in LSCMO-S are silicon and oxygen. The other elements are dispersed exactly in sociometric amounts. GO is mostly composed of carbon atoms and a percentage of oxygen with no other trace elements. Hence, the synthesized material is pure. LSCMO-S/GO (containing 10% LSCMO-S) showed a high amount of Carbon with sociometric amounts of La, Sr, Mn, and Cu.

#### 3.1.3. Raman Spectroscopy

**Figure 3:**
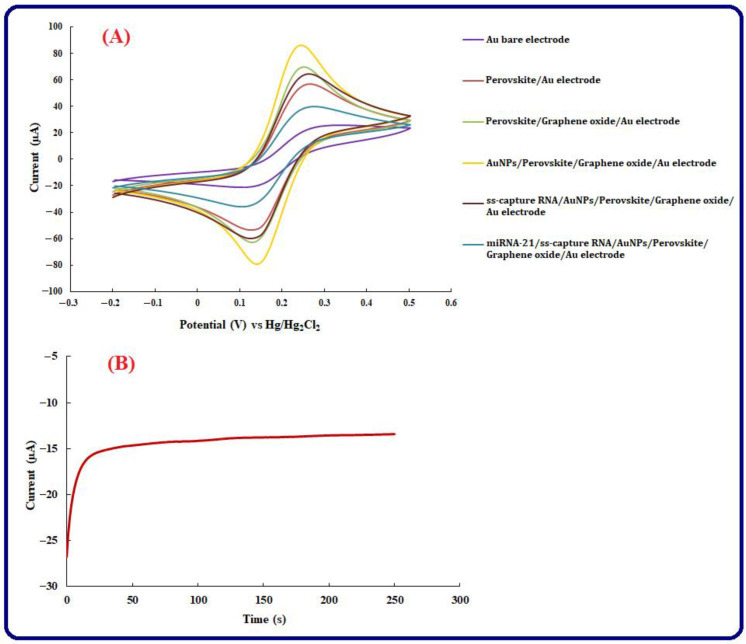
(**A**) CV voltammograms of Au bare electrode, Au modified electrode by Perovskite, Au modified electrode by Perovskite/graphene oxide, Au modified electrode by Perovskite/graphene oxide/AuNPs/ss-capture RNA, and Perovskite/graphene oxide/AuNPs/ss-capture RNA/miRNA-21 in [Fe(CN)_6_]^3−/4−^ as a redox probe. Also, the scan rate is 50 mV s^−1,^ and the potential range is −0.2 to +0.5 V, (**B**) Au electrode Chronoamprogram in the solution including 20 mL of 0.01 M HAuCl_4_ for immobilizing of AuNPs at E = 0 V for 500 s.

Raman spectroscopy was used to determine the structural changes of the prepared samples (LSCMO-S, GO, and LSCMO-S/G; Figure 3C). At GO, two strong peaks are seen at 1354 cm^−1^ (referenced to the D mode), indicating the presence of sp_3_ defects, and 1621 cm^−1^ (referenced to the G mode), associated with all sp_2_ carbon forms [59]. The vibrational mode of the perovskite material (ABO_3_) is active in the 200 cm^−1^ region [60]. The characteristic vibrational band of SiO_2_, centered around 143 cm^−1^, can also be observed in LSCMO-S [61]. Compared to the pure LSCMO-S, the intensity of the peaks of the LSCMO-S hybrid GO has slightly decreased, and some of the main peaks corresponding to LSCMO-S have disappeared due to the high content of GO in the hybrid sample.

#### 3.1.4. X-ray Powder Diffraction (XRD)

Figure 2D illustrates the XRD patterns of the samples (LSCMO-S (a), LSCMO-S/GO (b), and GO (c)). For LSCMO-S, all of the diffraction peaks of LSCM, which are consistent with the pattern reported for ABO_3_ (JCPDS No. 75–2100), are observed. Also, the silica content embedded in the LSCM matrix can be seen as segregated phases. Moreover, in the SiO_2_ spheres, a broad peak centered at 2*θ* = 20.5° is observed. For the calculation of the average size of SiO_2_ spheres, the Scherrer equation (Equation (1)) was applied:

(1) $$L=\frac{K\lambda }{BCos\left(\theta \right)}$$

In Equation (1), *L* is the average crystallite size, *K* is the Scherrer constant (1), *λ* represents the X-ray wavelength (0.15406 nm), *B* represents the line-broadening full-width at half maximum, *θ* represents the Bragg diffraction angle. Here, 2*θ* = 22.5° was selected to calculate the average crystallite size of the SiO_2_. The average crystallite size of the SiO_2_ is 35.86 nm. XRD pattern of GO shows three peaks that peak at 26.44° and 42.6° due to the presence of graphite [35]. For the composite of the GO and perovskite (10/1), the intensity of the diffraction peaks of the perovskite slightly decreased, and some of the main peaks corresponding to the LSCM/SiO_2_ disappeared due to the high content of GO.

### 3.2. Electrochemical Investigation of the Engineered Genosensing Assay

Electrochemical behavior The CV method in a solution containing [Fe(CN)_6_]^3−/4−^ has been used to assess electrochemical behavior associated with the modification of the Au electrode. CVs of the AuNPs/perovskite/graphene oxide/Au electrode, perovskite/graphene oxide/Au electrode, and bare Au electrode are represented in Figure 3A. Accordingly, different electrochemical performances in terms of potential and current are considered for Au electrode modified by ss-miRNA (miRNA-21/ss-capture RNA/AuNPs/perovskite/graphene oxide/Au electrode) and Au electrode modified by ss-capture RNA (ss-capture RNA/AuNPs/perovskite/graphene oxide/Au electrode). The peak current of 21.7 μA at 0.25 V is represented by the obtained voltammograms associated with the bare Au electrode. It was possible to detect the oxidation peak with a current of 56.5 μA at a potential of 0.23 V after modifying the Au electrode surface by applying perovskite/graphene oxide. The outcome represents that the peak current can be enlarged in the presence of perovskite/graphene oxide as an effective modifier and its reason is the presence of the various size pores on their surface and large surface area. According to Figure 3B, a good surface for probe immobilization is provided by the electrodeposition of AuNPs by the CA method, and according to CV patterns, the electrochemical response (0.23 V, 70 µA) can be enhanced by the deposition of AuNPs. A slight decrease in the current of the oxidation peak has been noticed, and the peak potential became 0.25 V after immobilizing ss-capture RNA. Perhaps, the self-assembly binds among thiol groups at the ends of ss-capture RNAs, and Au elements can provide such an alteration, which could happen on the modified Au electrode surface. After the fabrication of the genosensor, miRNA-21 can be added, and after the occurrence of hybridization, the peak current decreased to 39.4 μA. The associated result represents that efficient miRNA-21 immobilization on the substrate was performed.

### 3.3. ss-Capture RNA Immobilization Time and Hybridization Time

**Figure 4:**
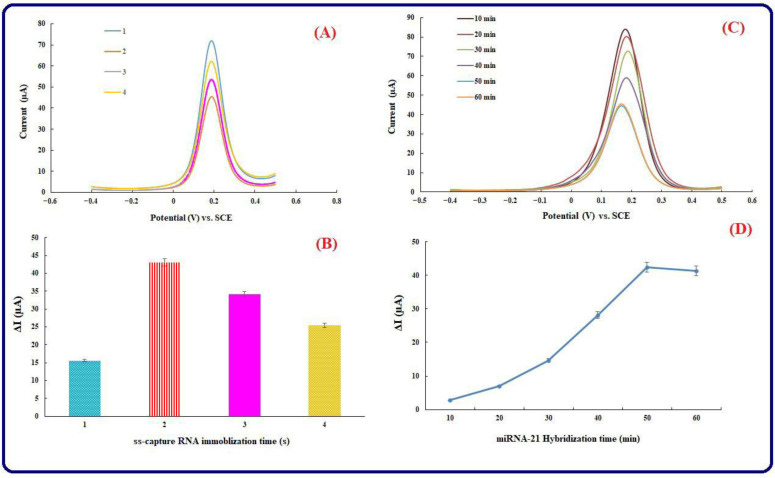
(**A**) DPV curves of ss-capture RNA immobilization time (10–120 min at 30-min intervals) on the surface of the gold electrode, (**B**) Histogram for ss-capture RNA immobilization time, (**C**) DPV voltammograms of the fabricated genosensor after incubation with miRNA-21 in different times (10–60 min at 10-min intervals). [Fe(CN)_6_]^3−/4−^ is applied as a redox probe, ss-capture RNA concentration is 1 µM, and also the scan rate is 50 mV s^−1^, (**D**) Histogram of miRNA-21 incubation time. Equilibration time = 0, E initial = −0.2 V, E end = 0.5 V, Estep = 0.1 V, frequency = 10 Hz, and amplitude = 0.01995 V.

The ss-capture RNA immobilization time has a drastic effect on the determination of miRNA-21, efficiency, hybridization, and sensitivity of the genosensing assay. In this study, the immobilization time of ss-capture RNA has been investigated for 1–4 h to reach the optimal time for the best performance of the biosensing platform. As shown in Figure 4A,B, increasing immobilization time from 1 to 2 h causes to increase in the related peak currents as well. Subsequently, the electrochemical responses were reduced with a steady increase in immobilization time.

On the other hand, one of the most significant steps is considered to be the optimization of the target hybridization time. Therefore, a 10-μL (0.1 nM) miRNA-21 has been dropped on the surface of the Au-modified electrode. The hybridization duration can have an important role in the research. Therefore, optimum conditions have been selected to examine different miRNA-21 incubation times. Times of 10–60 min have been used to incubate the genosensing assay by miRNA-21 at 10-min intervals at 25 °C (Figure 4C). Subsequently, an optimum time for target hybridization time has been obtained using the DPV method. After incubation at certain times and drying/washing the Au electrode surface, DPVs have been achieved in a supporting electrode solution including [Fe(CN)_6_]^3−/4−^. According to Figure 4D, it was possible to verify the greatest peak current associated with the process of hybridization at 50 min. Therefore, it was possible to obtain the hybridization of ss-capture RNA with miRNA-21 at an optimum incubation time of 50 min.

### 3.4. Kinetic Investigation

**Figure 5:**
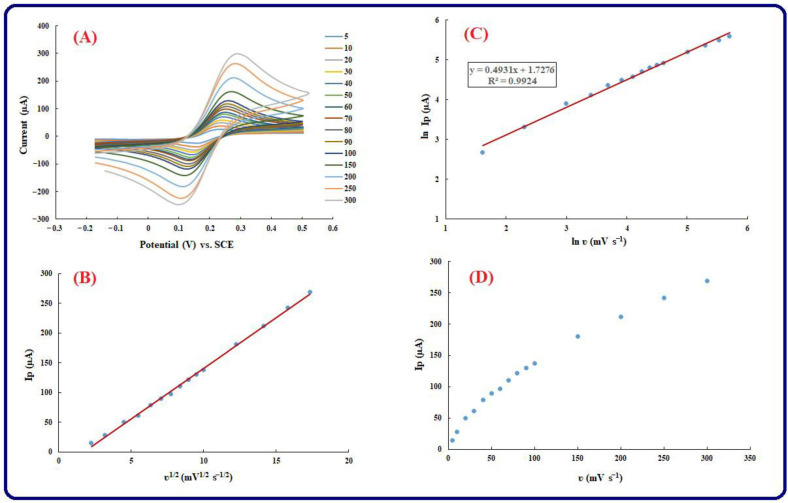
(**A**) CV voltammograms of Perovskite/graphene oxide/AuNPs modified Au electrode in the solution containing Fe(CN)_6_ ^3−/4−^, KCl in various scan rates (10, 20, 30, 40, 50, 60, 70, 90, 100, 150, 200, 250, 300 mVs^−1^): [Fe(CN)_6_]^3−/4−^ is applied as a supporting electrolyte, (**B**) Plot of *Ip* (µA) vs. v ^1/2^ (V^1/2^. s^−1/2^), (**C**) Ln *Ip* (µA) vs. Ln ʋ (mV s^−1^), (**D**) *Ip* (µA) vs. v ^1/2^ (mV^1/2^. s^−1/2^).

Figure 3 represents the cyclic voltammograms (CVs) of Au-modified electrodes by AuNPs/perovskite/graphene oxide achieved at different scan rates. The various scan rates from 1 to 1000 mV s^−1^ have been used to investigate the kinetic performance of the fabricated genosensing platform by the CV technique. According to Figure 5A, it is possible to increase the width of the voltammograms, and the anodic and cathodic peaks increase in greater scan rates. Such a fact verifies that, in the smaller scan rates, the electron exchange increases gradually on the electrode surface, and accordingly, thinner voltammograms with smaller peak currents are resulted (Figure 5A). Two significant aspects are considered for the dependence of *Ip* vs. *ʋ*^1/2^ or and ln *Ip* vs. ln *ʋ* to estimate the reversibility of a reaction and present the control of the reaction by adsorption or diffusion. According to Figure 5B, the linear dependence (R^2^ = 0.996) of the anodic and cathodic peak currents with square roots of scan rates is represented by the scan rate analysis. Furthermore, the linear dependence (R^2^ = 0.9829) of Ln *Ip* vs. Ln in the chosen range of scan rates (i.e., 1–300 mV s^−1)^ is confirmed by Figure 5C. Such results confirm the control of mass transfer through the diffusion process. Consequently, it is possible to say that the Randles–Sevcik equation is followed by the electrochemical process:

*Ip* = 2.69 × 10^5^ *AD*^1/2^ *n*^3/2^*C υ*^1/2^ (2)

According to Equation (2), the peak current (μA) is represented by *Ip*, the electroactive surface (cm^2^) is represented by *A*, *D* represents the molecular diffusion coefficient in solution (cm^2^/s), the electron is represented by *n*, which is transferred in an electrochemical reaction, relates to the scan rate (V/s), and the concentration of the analyte (mol/dm^3^) is represented by *C*. Because of such a fact that as a result of the change in scanning velocity, the peak potential partially alters, the reaction is irreversibly controlled by the redox process. On another hand, to explain the mechanism of mass transfer, Figure 5C represents the equivalence of the slope with 0.5, and such an outcome represents that the electrochemical reaction is diffusional control. A slope near 1 and 0.5 verifies the control of the electrochemical procedure through adsorption and diffusion, respectively. In conclusion, Figure 5D represents that the dependence of *Ip* vs. does not have a linear pattern and verifies that there is not a linear relation between *Ip* vs. *ʋ*. Afterward, there is no adsorption dependence between the electrochemical reaction. Additionally, CV with 5 mM [Fe(CN)_6_]^3−/4−^ as a probe at various scan rates was used to obtain the surface area. To calculate the surface area, the mentioned Randles–Sevcik (Equation (1) has been applied. Anodic peak current is represented by *Ip*, *n* referred to the electron transfer, is equal to 1, the concentration of [Fe(CN)_6_]^3−/4−^ is represented by *C*0, the scan rate is represented by *ν*, and *D* is equal to 0.76 × 10^−5^ cm^2^s^−1^ which presents the diffusion coefficient. Based on the slope of *Ip* and *ν*^1/2^ relation, 0.073 cm^2^ has been obtained for the surface area. Based on the cyclic voltammograms of Figure 5, an increase in the peak currents is based on an expansion of the modified electrode surface. In comparison to the bare electrode, the rest of the electrodes have an increase in surface area, but the increase in surface area of the desired electrode is higher than that of others. The order of electrode surface areas related to their voltammograms of CV is in the following manner: AuNPs/perovskite/graphene oxide/Au electrode > perovskite/graphene oxide/Au electrode.

### 3.5. Calibration Plot and Analytical Features

**Figure 6:**
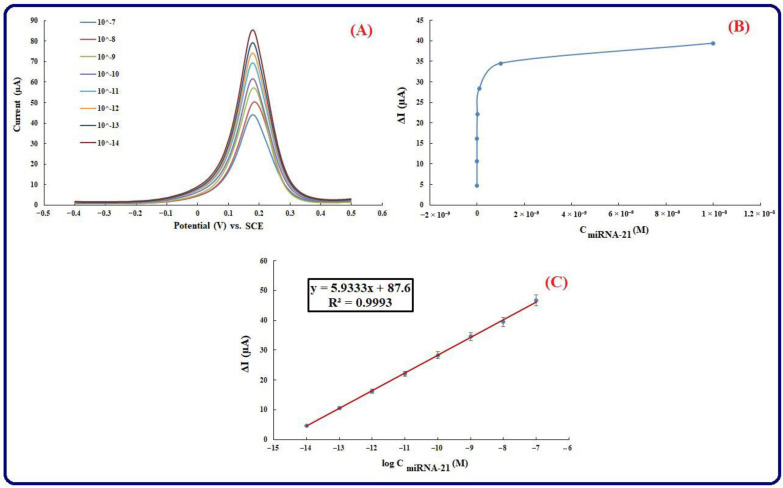
(**A**) DPVs (Equilibration time = 0, E initial = −0.2 V, Estep = 0.1 V, E end = 0.5 V, Amplitude = 0.01995 V, frequency = 10 Hz) of the fabricated genosensor in various concentrations of miRNA-21 include: (10^−14^–10^−7^ M). Supporting electrolyte is [Fe(CN)_6_]^3−/4−^, [Fe(CN)_6_]^3−/4−^ is applied as a redox probe, ss-capture RNA concentration is 1 µM, (**B**) Dependency of peak currents different concentrations (Calibration plot), (**C**) Plot of Ln C (concentrations).

To evaluate the analytical features of RNA-based genosensing assay, the sensitivity of a fabricated genosensing bio-assay can be a particular parameter. Therefore, the incubation of different concentrations of miRNA-21 has been used to measure the rate of the hybridization process at 25 °C for 50 min. Figure 6A represents the results associated with DPV of different miRNA-21 concentrations (10^−14^–10^−7^ M at 10^−1^ M intervals). The outcomes represent that an increase in the concentration of miRNA-21 leads to a decrease in the peak current. [Fe(CN)_6_]^3−/4−^ and RNA have negative charges, and this is the reason for a decrease in the current. When casting ss-capture RNA on the Au electrode surface, the negative charges associated with the RNA and solution, including [Fe(CN)_6_]^3−/4−,^ have an electrostatic repulsion. After adding miRNA-21, accumulating the negative charge on the electrode is enlarged, and therefore, an increase in the electrostatic repulsion and a reduction in the electrochemical signal have resulted. Accordingly, the non-occurrence or occurrence of the hybridization process is diagnosed by applying the [Fe(CN)_6_]^3−/4−^ to record the electrochemical signal. More significantly, a desirable linear relation (R^2^ = 0.9992) between the DPV peak current (ΔI = Iss-capture RNA–ImiRNA-21) and the logarithm values of miRNA-21 concentration is observed (Figure 6B). According to the optimum conditions, 10^−14^ to 10^−7^ has been obtained for LR (linear range). Furthermore, LOQ (limit of quantification) and LOD (limit of detection) have been at 8.75 fM and 2.94 fM, respectively. It is noteworthy that supplying the limit of quantification and limit of detection of the fabricated genosensing assay is based on the 10 σ/m and 3 σ/m criteria, respectively (where the standard deviation of the intercept or the standard deviation of the blank is represented by σ and the slope of the calibration plot is represented by m). Therefore, the fabricated genosensing bio-assay has been used to determine miRNA-21 with high sensitivity. It was possible to perform all the measurements in triplicates, and the relative standard deviations of the measurements (RSD% = 3.4–5.3) have been represented by the error bars, which demonstrates the great accuracy of the genosensor. Based on the explanations concerning the earlier methods, it is said that all analytical features have improved. In this research, LOQ and LOD are significantly smaller, which represents the great sensitivity of the presented genosensing assay. The high electron transfer rate provides high sensitivity, and applying AuNPs/perovskite/graphene oxide nanocomposite provides increased surface area. Additionally, the presented genosensor has a wide linear range, and it can be used to analyze the wide concentration of the analytes. The achieved values represent that a suitable method for miRNA-21 determination at low concentrations with a wide linear range is offered by the engineered platform (Table 2).

### 3.6. Selectivity Investigation

**Figure 7:**
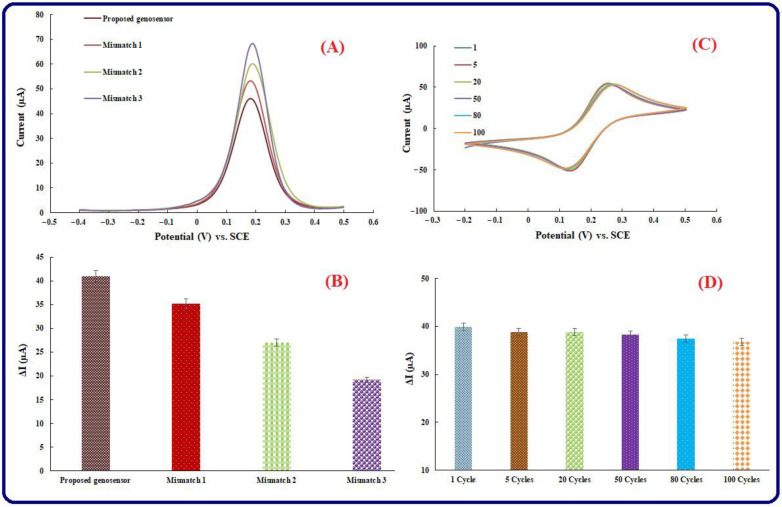
(**A**) Selectivity of the fabricated genosensor: DPVs of the engineered genosensor in hybridization by miRNA-21, 1-mismatch miRNA-21, 2-mismatch, and 3-mismatch miRNA-21. The scan rate is 50 mV s^−1^. (**B**) Histograms of the proposed genosensing assay selectivity, (**C**) CVs of modified Au electrode in different cycle numbers. (**D**) Histogram associated with different cycle numbers.

According to the research, mismatched RNA sequences have been used to assess the selectivity of the planned bio-assay. To investigate the aforementioned step, 10.0 μL of miRNA-21 and the mismatch sequences with 25 μM concentration have been hybridized with the ss-capture probe on the surface of the presented genosensing bio-assay and incubated at 25 °C after doing the preparation steps and fabricating the required genosensor. Table 1 represents the sequences of the mismatches. According to Figure 7A,B, due to the successful and effective miRNA-21 hybridization process, the peak current respecting the presented bioassay is smaller than the other mismatch RNA sequences. In comparison to the mismatch target sequence, the results demonstrate the great selectivity of the electrochemical response associated with mismatch 1 is close to the electrochemical response of the presented genosensing bio-assay and smaller than other mismatches, and the reason is that the existence of similarity between the sequence of our miRNA-21 probe and the sequences of the related mismatch 1 sequence. Accordingly, due to the abovementioned reason, the electrochemical response of mismatch 2 and 3 have been increased, and their electrochemical signal is close to the ss-capture RNA-related signal. In another hand, negative control tests have been used to investigate the selectivity of the fabricated genosensor. Instead of miRNA-21, miRNA-126 as negative control has been used for the hybridization process. Therefore, there is one more rational reason for the confirmation of the great selectivity of fabricated genosensing bio-assay.

### 3.7. Repeatability, Reproducibility, and Stability of Genosensing Bio-Assay

The performance of the proposed genosensing assay is related to the stability of RNA probes in electrochemical reactions that happen throughout the genosensor fabrication process. Investigating the CV signals on the electrode in a supporting solution including [Fe(CN)_6_] ^3−/4−^ can help assess the stability of the genosensor based on the mentioned modifiers (AuNPs/perovskite/graphene oxide). Different cycle numbers, including 1, 5, 20, 50, 80, and 100, have been used to perform this step. According to Figure 7C,D, significant substantial alteration is not seen between the 100 cycles and the first one. In another word, constancy and reliability are seen among the verified cycles. The results represent a suitable stability level in the well-fabricated genosensing assay.

In another hand, to examine the genosensing assay reproducibility, 5 different modified electrodes have been arranged by ss-capture RNA/AuNPs/perovskite/graphene oxide to determine 0.1 nM of the miRNA-21, and thus, it was possible to efficiently measure the electrochemical responses. RSD (relative standard deviation) of five modified electrodes was equal to 2.9%. Five determinations of 0.1 pM miRNA-21 with one modified electrode have been used to investigate the repeatability of the fabricated genosensing assay. RSD was equal to 4.7%. Satisfactory values for the novel analytical genosensor have been demonstrated by RSDs of the achieved electrochemical responses for reproducibility and repeatability steps.

### 3.8. Investigating the Performance of Bioassays in Real Samples

#### 3.8.1. Gastric Cancer Cell Lines

**Figure 8:**
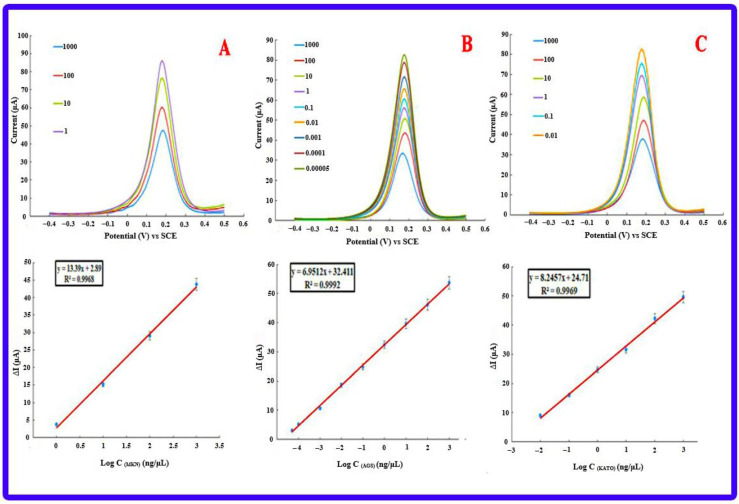
Electrochemical results of detecting different concentrations of miRNA-21 in cell lines (**A**) MKN-45 with a detection limit of 1 ng/µL, (**B**) AGS with a detection limit of 5 × 10^−5^ ng/µL and (**C**) KATO III with a detection limit of 0.01 ng/µL.

**Figure 9:**
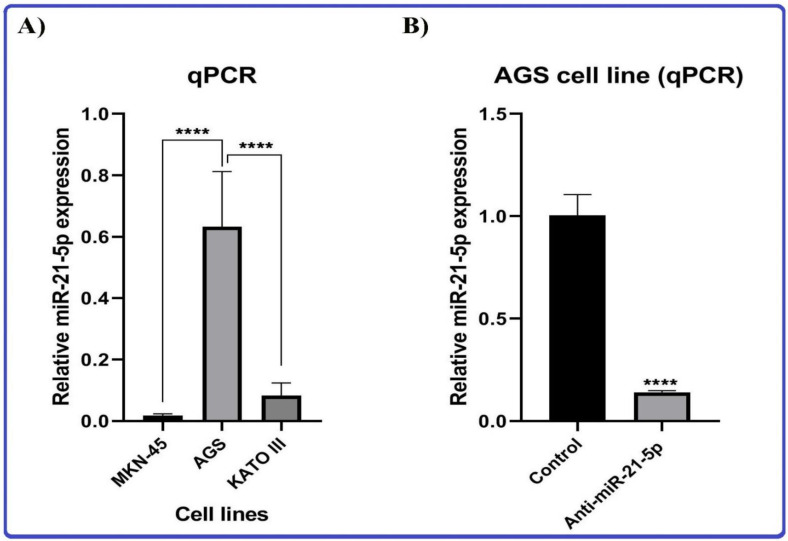
(**A**) Relative miRNA-21 expression in MKN-45, AGS, and KATO III cell lines by qRT-PCR. (**B**) Relative miRNA-21 expression in AGS cell line treated with Anti-miR-21 and control cell line by qRT-PCR. **** indicates *p* value < 0.0001.

To evaluate the efficiency of the designed biosensor, three gastric cancer cell lines, including AGS, KATO III, and MKN-45, were selected. First, the selected cell lines were cultured, and then their RNA was extracted. The investigation of RNA samples from all three cell lines by the designed electrochemical genosensor started with the DPV method from a base concentration of 1000 ng/μL and reached the lowest detectable concentration with step-by-step dilution similar to calibration. The minimum concentrations detected for AGS, KATO III, and MKN-45 cell lines were 5 × 10^−5^ ng/μL, 0.01 ng/μL, and 1 ng/μL, respectively (Figure 8). In addition, the expression level of miRNA-21 in all three cell lines was checked by the qRT-PCR technique, and the expression of miRNA-21 in the AGS cell line was significantly higher than in other lines. Figure 9A confirms the high expression of miRNA-21 in the AGS cell line.

**Figure 10:**
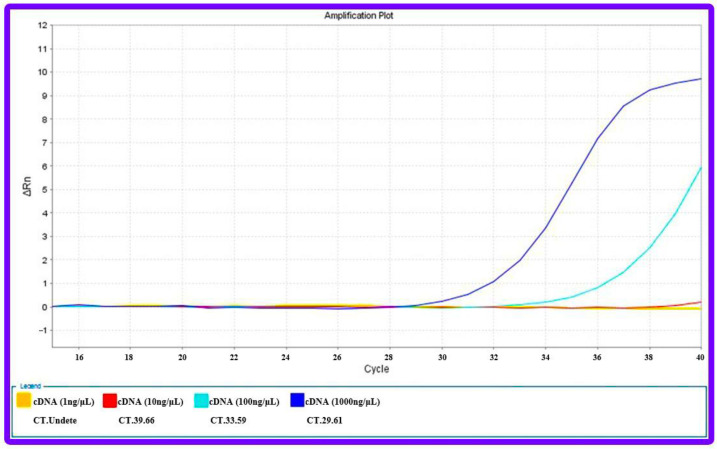
qRT-PCR results for different concentrations of RNA sample extracted from AGS cell line.

To compare the detection ability of the qRT-PCR technique with the designed electrochemical biosensor, different concentrations of extracted RNA from AGS cell lines and cDNAs, which were synthesized for the qRT-PCR technique, were prepared. Subsequently, the investigation process of the proposed genosensing assay has been performed. Based on the results, there was not enough synthesis signal in the qRT-PCR technique for concentrations less than 100 ng/μL (Figure 10). At the same time, the electrochemical biosensor detected up to 5 × 10^−5^ ng/μL concentration (Figure 9A).

#### 3.8.2. Anti-miR-21 Treatment

**Figure 11:**
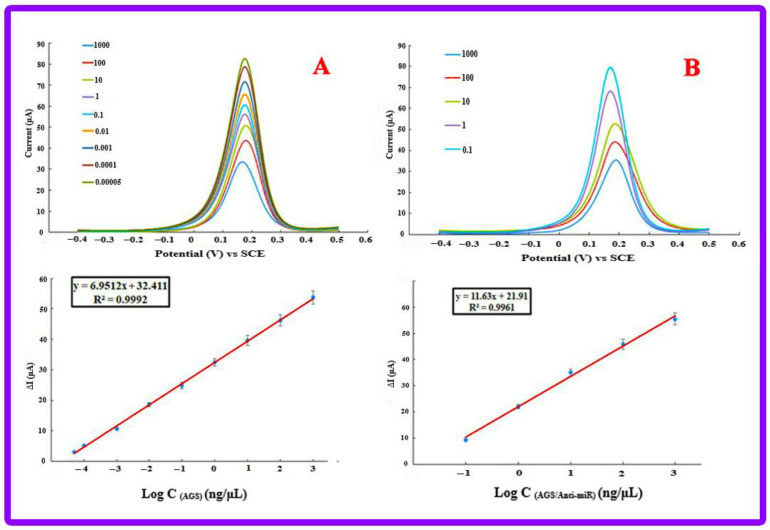
Electrochemical results of miRNA-21 measure changes in AGS cell line before and after treatment with Anti-miR-21. (**A**) The control group with a limit of detection 5 × 10^−5^ ng/μL and the (**B**) group treated with Anti-miR-21 with a limit of detection 0.1 ng/μL.

To check the specificity and accuracy of the designed genosensor, the AGS cell line was treated with anti-miR-21 and subsequently analyzed by qRT-PCR technique. Accordingly, treatment with anti-miR-21 leads to a decrease in miRNA-21 expression (Figure 7B). The electrochemical investigation was also done by the fabricated genosensor, and the minimum detectable dilution was obtained as 0.1 ng/μL, which has a significant decrease compared to the state before treatment with Anti-miR-21 (5 × 10^−5^ ng/μL) (Figure 11).

Different analytical features of the genosensor have been efficiently improved (Table 2) when comparing this investigation with other research works. The great efficiency of our fabricated genosensor is represented by these results. The better performance of our genosensing assay is confirmed by investigating various parameters such as selectivity, stability, regeneration, repeatability, and reproducibility. Observing that biosensing and sensing assays provide a new field of study, maintaining the newness of each research work, and synthesis of the novel composite as an effective modifier is a difficult task.

## 4. Conclusions, Challenges, and Future Outlooks

In conclusion, it was possible to develop a new label-free electrochemical genosensing assay based on RNA hybridization for sensitively determining miRNA-21 in real samples. According to the achieved results, it was possible to confirm that such a high-sensing bio-assay has the capability of being applied in recognizing varieties of miRNAs in clinical samples. With the comparison of the outcomes of the reported investigations and the analytical features of the fabricated genosensing assay, it is concluded that the presented bio-assay has superior sensitivity to others. In comparison to other approaches, a singular RNA-based bio-assay with higher characteristics for determining miRNAs is developed by using such a methodology. A high potential of selecting the target from one-, two-, and three-base mismatched sequences are represented by the well-fabricated genosensing assay. In another hand, the current investigation has been used to propose a selective bio-device for sensitively determining miRNA-21 in the existence of negative control. Additionally, the reusability and regeneration of the well-aligned bio-assay have been researched. For identifying the critical biomarkers and timely administrating efficient treatment to patients, the promising aspect of fabricated genosensing assay is its singular characteristics. In conclusion, such an investigation can be a new method to develop such genosensing bio-devices for the greatly selective and sensitive determination of other significant biomarkers.

## Figures and Tables

**Table 1:** Oligonucleotide sequences of ss-capture RNA (capture probe), miRNA-21(target), mismatch (1, 2, 3), negative control (miRNA-126).

Oligonucleotide	Sequences
**Capture probe**	5′-SH-(CH20)6-UCAACAUCAGUCUGAUAAGCUA-3′
**miRNA-21**	5′-UAGCUUAUCAGACUGAUGUUGA-3′
**Mismatch1**	5′-UAGCUUAUCA**C**ACUGAUGUUGA-3′
**Mismatch2**	5′-UAGCUUAU**A**AGACU**A**AUGUUGA-3′
**Mismatch3**	5′-U**U**GCUUAUC**G**GACUGAU**C**UUGA-3′
**miRNA-126**	5′-UCGUACCGUGAGUAAUAAUGCG-3′

**Table 2:** Comparison of fabricated genosensor with other related methods for highly sensitive recognition of miRNA-21.

Methods	Modifier Agents	Linear Range	LOD	References
ECL ^a^	GCE/Au NPs/DNA tweezer/report DNA	0.02–150 pM	6.3 fM	[62]
SPR ^b^	Au/MB/miRNA/StreGNRs	0.1–100 pM	45 fM	[63]
Fluorescence	Au nanorod functionalized polydiacetylene microtube waveguide	50–1000 fM	10 fM	[64]
EC ^c^	miRNA-21/DNA-Au NPs@MoS_2_	0.01–1000	0.78 fM	[65]
Colorimetric	-	10 pM–1 nM	1 pM	[66]
LFNAB ^d^	AuPt NF-based	2–5000 pM	0.3 pM	[67]
Electrochemical	AuNPs/GQDs/GO	0.001–1000 pM	0.04 fM	[68]
Electrochemical	AuNPMMBs/MAuE	5–600 fM	0.20 fM	[69]
Electrochemical	TDN/depAu/GCE	10 µM–10 nM	18.9 aM	[70]
Ratiometric electrochemistry	Mg^2+^-dependent DNAzyme-cleavage cycling	10 fM–0.1 nM	1.8 fM	[71]
THz ^e^ spectroscopy	THz metamaterials	1 fM–10 pM	14.54 aM	[72]
Electrochemical genosensor	Perovskite-graphene oxide	10 fM–100 nM	2.94 fM	**The present study**

^a^ Electrochemiluminescence; ^b^ Surface plasmon resonance; ^c^ Electrochemistry; ^d^ Lateral flow nucleic acid biosensor; ^e^ Terahertz.

## Data Availability

The data presented in this study are available on request from the corresponding author.
